# Supplementary material for: Elucidating the Molecular Mechanisms by which Seed-Borne Endophytic Fungi, Epichloë gansuensis, Increases the Tolerance of Achnatherum inebrians to NaCl Stress
Source: Int J Mol Sci. 2021 Dec 7;22(24):13191. doi: 10.3390/ijms222413191 (PMC8706252; doi:10.3390/ijms222413191)
Supplement: Supplementary file 1 [file ijms-22-13191-s001.zip › ijms-1421862-supplementary.pdf]

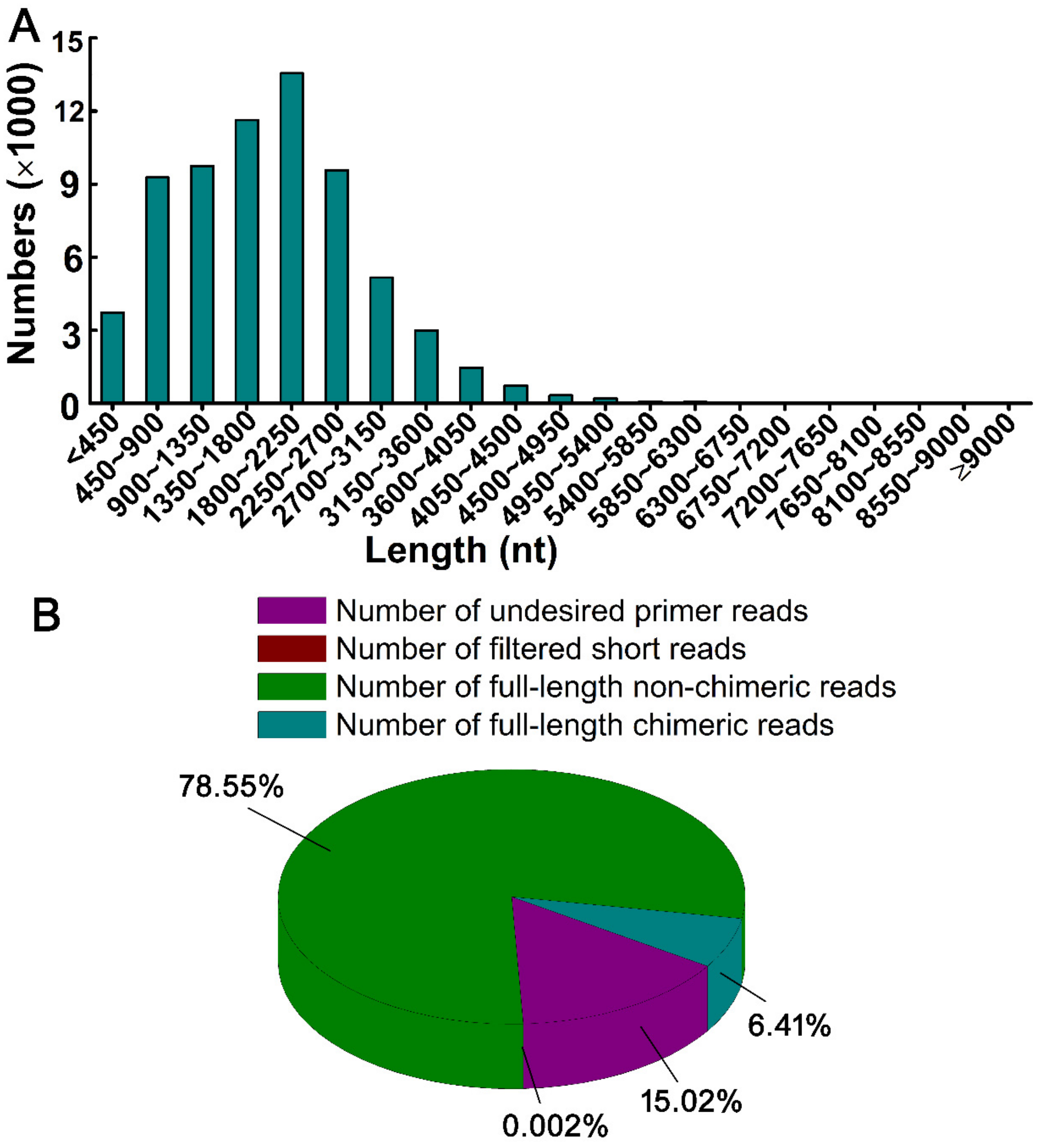

Supplementary Figure S1. The outline of PacBio RS II single-molecule real-time (SMRT) sequencing in *A. inebrians*. (A): The length distributions and numbers of 940,319 reads from PacBio libraries. (B): Proportion of different types of PacBio reads.

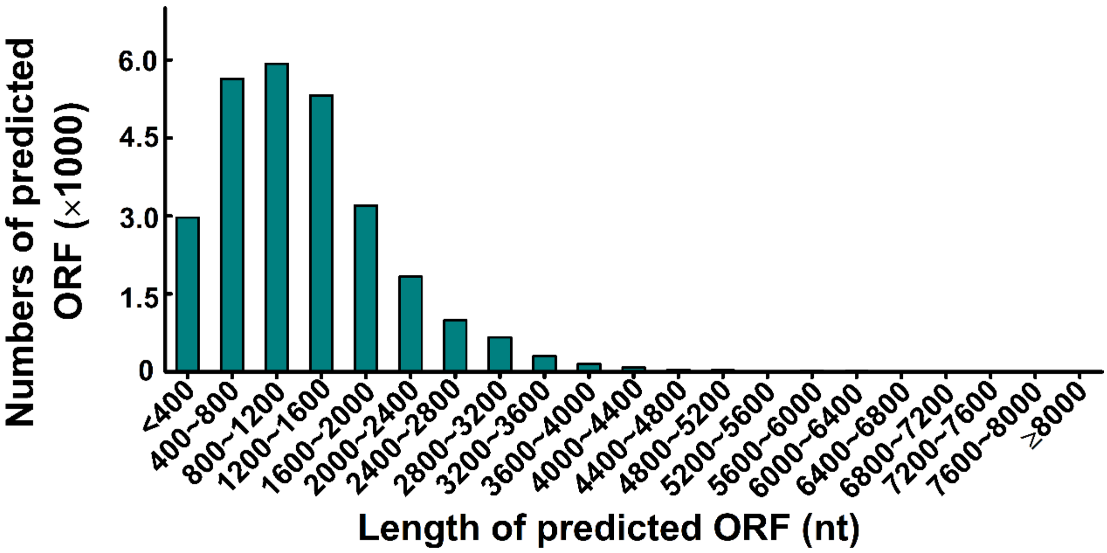

Supplementary Figure S2. The length distributions and numbers of open reading frame (ORF) of 36,105 non-redundant full-length transcripts in *A. inebrians*.

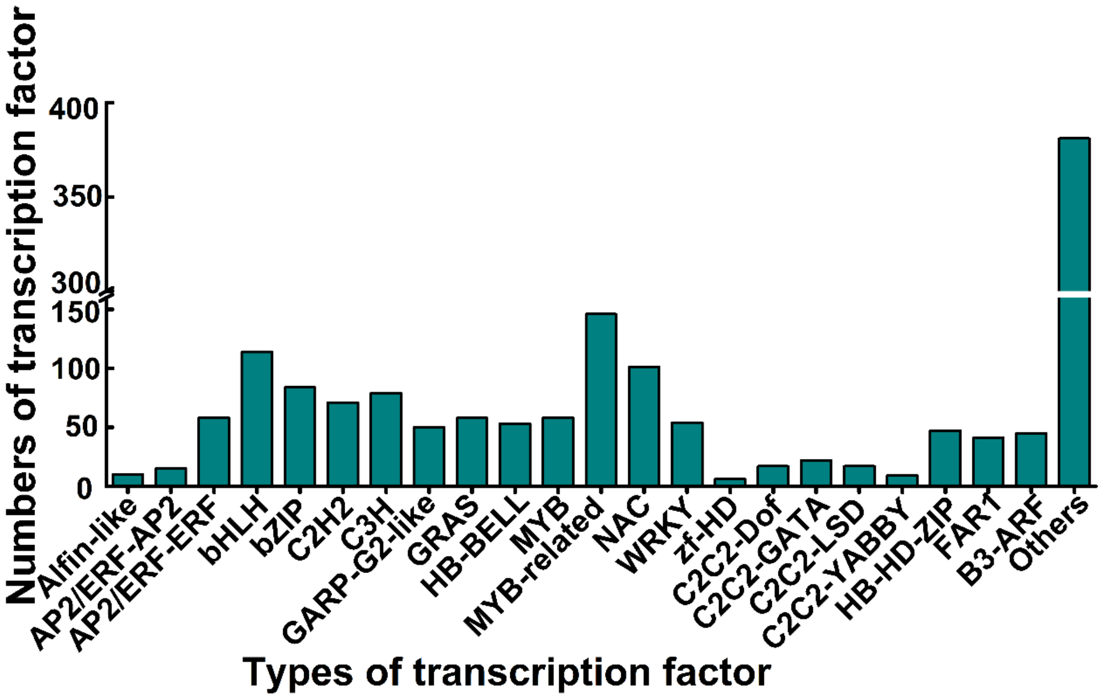

Supplementary Figure S3. The types and numbers of transcription factors of 36,105 non-redundant full-length transcripts in *A. iebrians*.

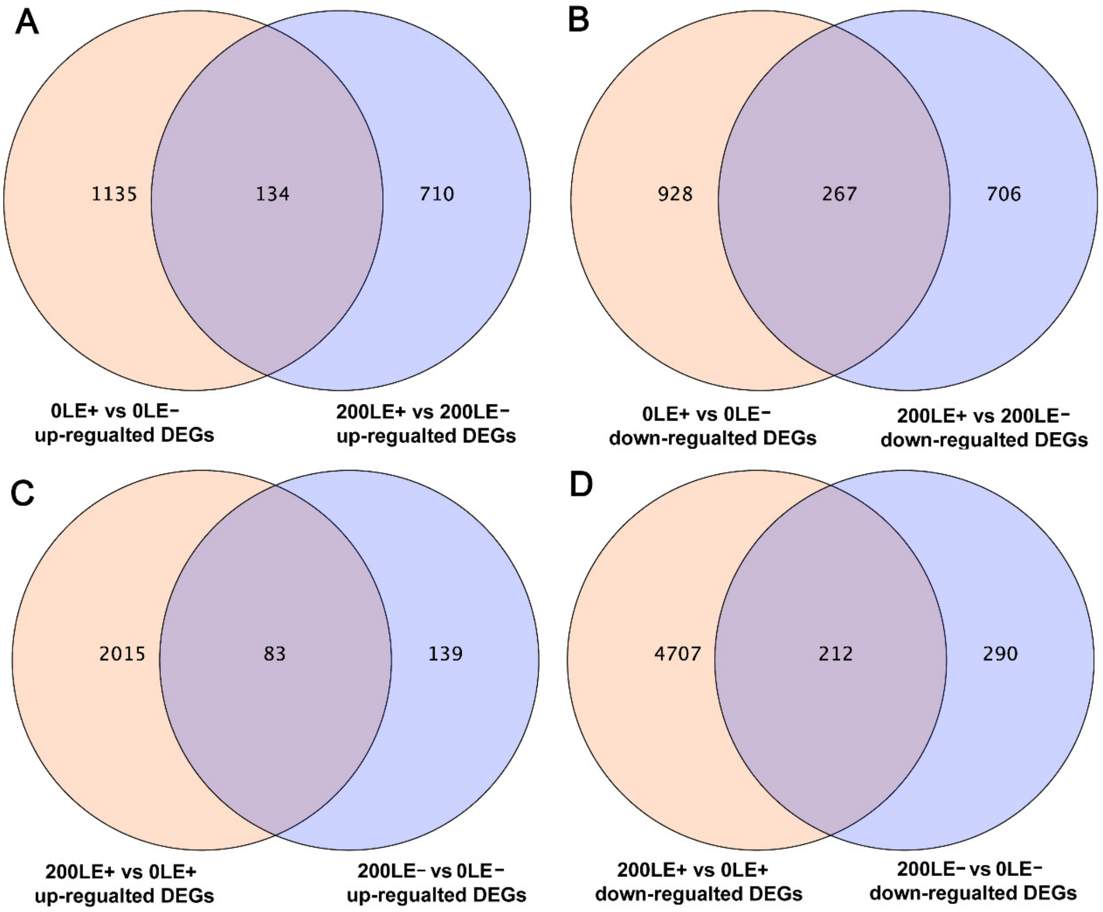

Supplementary Figure S4. Venn diagram demonstrating the non-overlapping and overlapping up- and down-regulated DEGs among the comparisons. (A): the up-regulated DEGs between (0LE+ vs 0LE−) and (200LE+ vs 200LE−); (B): the down-regulated DEGs between (0LE+ vs 0LE−) and (200LE+ vs 200LE−); (C): the up-regulated DEGs between (200LE+ vs 0LE+) and (200LE− vs 0LE−); (D) the down-regulated DEGs between (200LE+ vs 0LE+) and (200LE− vs 0LE−).

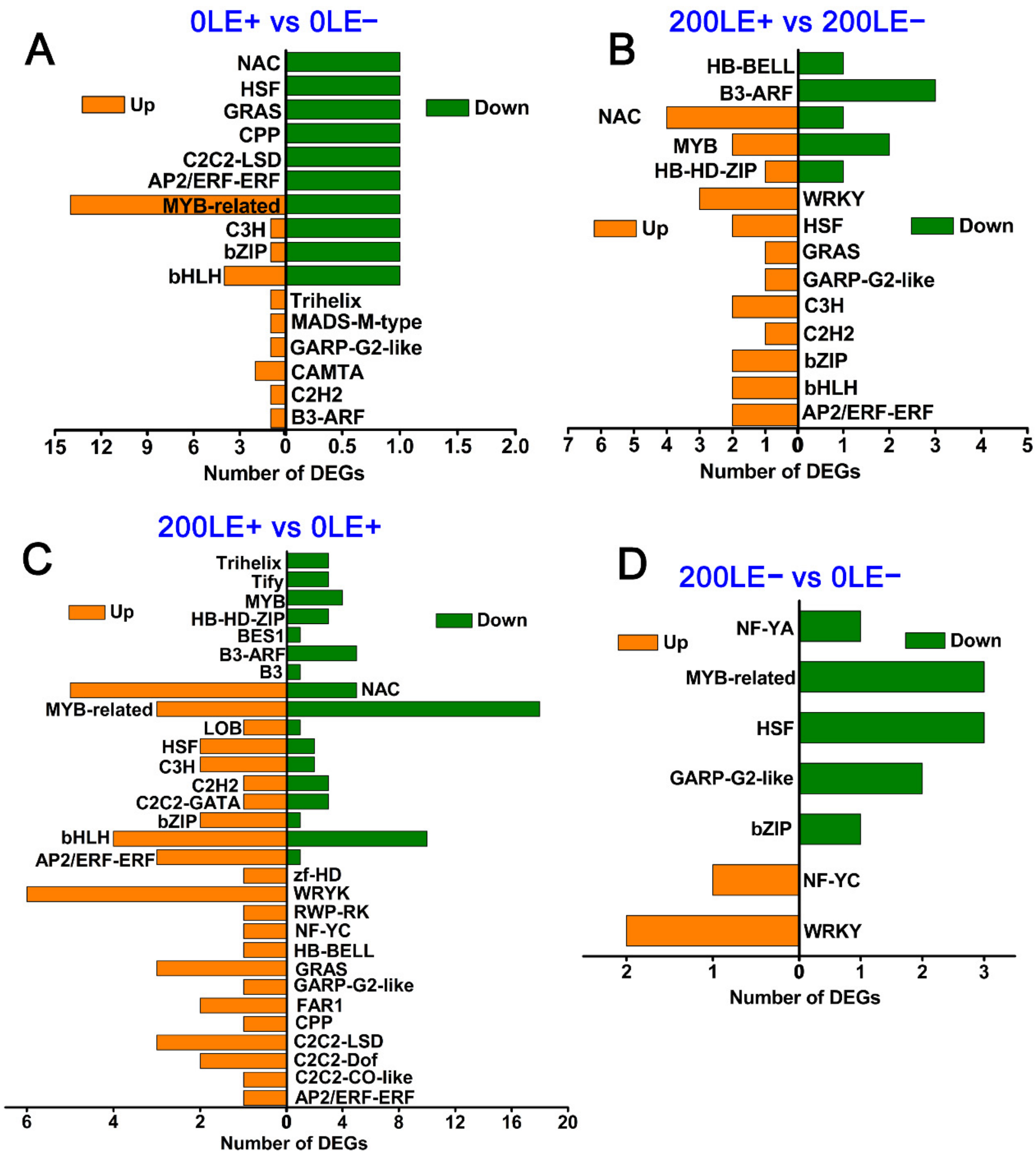

Supplementary Figure S5. The transcription factors of up- and down-regulated DEGs between 0LE+ vs 0LE− (A); between 200LE+ vs 200LE− (B); between 200LE+ vs 0LE+ (C); between 200LE− vs 0LE− (D).

Supplementary Table S1. Summary of PacBio-based RNA sequencing.

	**Number**
Reads of insert of PacBio sequencing	940,319
Bases of insert of PacBio sequencing (bp)	1,789,709,871
Number of undesired primer reads	141,272
Number of filtered short reads	142
Number of full-length non-chimeric reads	738,588
Number of full-length chimeric reads	60,317
Number of consensus isoforms	68,878
Average consensus isoforms read length	1,832
Number of polished high-quality isoforms	68,615
Number of polished low-quality isoforms	224

Supplementary Table S2. Prediction of simple sequence repeats (SSRs) out of our transcript datasets.

	**Number**
Total number of sequences examined	34702
Total size of examined sequences (bp)	68475072
Total number of identified SSRs	15945
Number of SSR containing sequences	11653
Number of sequences containing more than 1 SSR	3185
Number of SSRs present in compound formation	1664
Mono-nucleotide	3213
Di-nucleotide	3700
Tri-nucleotide	8493
Tetra-nucleotide	371
Penta-nucleotide	81
Hexa-nucleotide	87

Supplementary Table S3. Annotation of our transcript datasets to public databases. COG, clusters of orthologous groups; GO, gene ontology; KEGG, kyotoencyclopedia of genes and genomes; Pfam, a large collection of protein families; Swiss-Prot, a well-annotated and manually checked protein database; TrEMBL, an automatically annotated protein database; NR, a NCBI non-redundant protein database.

Annotated databases	Isoform Number	Percentage
COG	15188	42.07%
GO	29623	82.05%
KEGG	14998	41.54%
KOG	20815	57.65%
Pfam	28805	79.78%
Swiss-Prot	24715	68.45%
eggNOG	33376	92.44%
NR	33878	93.83%
All	33998	94.16%

Supplementary Table S4. The types and numbers of transcription factors

TF types	TF members
Alfin-like	10
AP2/ERF-AP2	15
AP2/ERF-ERF	58
B3	13
B3-ARF	45
BBR-BPC	6
BES1	1
bHLH	114
bZIP	84
C2C2-CO-like	4
C2C2-Dof	17
C2C2-GATA	22
C2C2-LSD	17
C2C2-YABBY	9
C2H2	71
C3H	79
CAMTA	10
CPP	10
CSD	1
DBB	7
DBP	10
EIL	10
FAR1	41
GARP-ARR-B	6
GARP-G2-like	50
GeBP	9
GRAS	58
GRF	4
HB-BELL	53
HB-HD-ZIP	47
HB-KNOX	4
HB-other	17
HB-PHD	4
HB-WOX	2
HRT	2
HSF	26
LIM	6
LOB	5
MADS-M-type	4
MADS-MIKC	12
MYB	58
MYB-related	146

NAC	101
NF-X1	1
NF-YA	31
NF-YB	8
NF-YC	11
OFP	4
PLATZ	7
RWP-RK	25
S1Fa-like	3
SBP	19
SRS	1
STAT	2
TCP	12
Tify	25
Trihelix	25
TUB	23
ULT	1
VOZ	5
Whirly	5
WRKY	54
zf-HD	6
